# Nutritional Status at Admission and Clinical Outcomes in Hospitalized Patients with COVID-19: Results from the MODIeCOSe in Italy Study

**DOI:** 10.3390/nu18203378

**Published:** 2026-10-15

**Authors:** Carola Buscemi, Matteo Monami, Cristiana Randazzo, Anna Maria Barile, Benedetta Ragghianti, Chiara Davoli, Enrico Maria Trecarichi, Helen Linda Morrone, Bruno Tassone, Piero Colombrita, Carlo Cottone, Maria Elena Greggi, Paolo Sbraccia, Valeria Guglielmi, Frida Leonetti, Danila Capoccia, Alexis Elias Malavazos, Sara Basilico, Ferruccio Santini, Alessandro Antonelli, Nicola Viola, Carlo Torti, Silvio Buscemi

**Affiliations:** 1 Dipartimento di Promozione della Salute, Materno-Infantile, Medicina Interna e Specialistica di Eccellenza (PROMISE), University of Palermo, Piazza delle Cliniche 2, 90127 Palermo, Italy; carola.buscemi@unipa.it (C.B.); cristiana.randazzo@unipa.it (C.R.); piero.colombrita@unipa.it (P.C.); silvio.buscemi@unipa.it (S.B.); 2 Unit of Internal Medicine, V. Cervello Hospital, 90133 Palermo, Italy; 3 Diabetology and Metabolic Disease Unit, Careggi Teaching Hospital and University of Florence, 50134 Florence, Italy; matteo.monami@unifi.it (M.M.); b.ragghianti@gmail.com (B.R.); 4 Unit of Clinical Nutrition, Obesity and Metabolic Diseases, University Hospital Policlinico “P. Giaccone”, Piazza delle Cliniche 2, 90127 Palermo, Italy; 5 Infectious and Tropical Diseases Unit, Department of Medical and Surgical Sciences, “Magna Graecia” University—“Mater Domini” Teaching Hospital, 88100 Catanzaro, Italy; chiara.davoli93@gmail.com (C.D.); e.trecarichi@unilink.it (E.M.T.); helen.morrone@gmail.com (H.L.M.); tassonebruno89@gmail.com (B.T.); carlo.torti@unicatt.it (C.T.); 6 Department of Life Science, Health, and Health Professions, Link Campus University, 00165 Rome, Italy; 7 COVID Internal Medicine Unit, Petralia Sottana Hospital, ASP 6, 90127 Palermo, Italy; carlocottone@libero.it (C.C.); mariaelenagreggi@libero.it (M.E.G.); 8 Department of Systems Medicine, University of Rome Tor Vergata, 00133 Rome, Italy; sbraccia@med.uniroma2.it (P.S.); valeria.guglielmi@uniroma2.it (V.G.); 9 Internal Medicine Unit—Obesity Center, University Hospital Policlinico Tor Vergata, 00133 Rome, Italy; 10 Diabetes Unit, Department of Medical-Surgical Sciences and Biotechnology, Santa Maria Goretti Hospital, “La Sapienza” University of Rome, 04100 Latina, Italy; frida.leonetti@uniroma2.it (F.L.); danila.capoccia@uniroma1.it (D.C.); 11 Endocrinology Unit, Clinical Nutrition and Cardiometabolic Prevention Service, IRCCS Policlinico San Donato, 20122 Milan, Italy; alexis.malavazos@gmail.com (A.E.M.);; 12 Department of Biomedical, Surgical and Dental Sciences, Università degli Studi di Milano, 20122 Milan, Italy; 13 Endocrinology Unit, Department of Clinical and Experimental Medicine, University of Pisa, 56126 Pisa, Italy; ferruccio.santini@unipi.it (F.S.); nicolaviola92@libero.it (N.V.); 14 Department of Surgical, Medical and Molecular Pathology and Critical Area, University of Pisa, 56126 Pisa, Italy; alessandro.antonelli@unipi.it; 15 Department of Healthcare Surveillance and Bioethics, Section of Infectious Diseases, Catholic University of Sacred Heart, 00168 Rome, Italy; 16 Infectious Diseases Unit, Department of Medical and Surgical Sciences, Fondazione Policlinico Universitario ‘A. Gemelli’ IRCCS, 00133 Rome, Italy

**Keywords:** COVID-19, nutritional status, malnutrition, CONUT, Prognostic Nutritional Index, anthropometry, acute illness, prognosis

## Abstract

**Background/Objectives**: Poor nutritional status is common among hospitalized patients and may adversely affect the response to severe acute illness. We investigated the association between nutritional status at hospital admission and clinical outcomes in patients hospitalized with COVID-19 during the first two pandemic waves in Italy. **Methods**: This retrospective multicenter study included 1187 patients hospitalized with COVID-19 across nine Italian centers participating in the MODIeCOSe in Italia registry. Nutritional status was assessed using the Controlling Nutritional Status (CONUT) score, the Prognostic Nutritional Index (PNI), and available anthropometric measures. The primary outcome was a composite of in-hospital death or transfer to an intensive care unit (ICU). Multivariable logistic regression was used to assess the independent association between nutritional status and outcome. **Results**: Overall, 275 patients (23.2%) experienced the composite adverse outcome. CONUT score was higher in patients with an adverse than in those with a favorable outcome (4.4 ± 2.3 vs. 3.4 ± 1.8; *p* < 0.001), whereas PNI did not differ significantly. Patients with an adverse outcome also had lower body weight, BMI, waist circumference, and waist-to-height ratio, although anthropometric data were available only in subsets of the cohort. In the main multivariable model adjusted for admission PaO_2_/FiO_2_ and relevant clinical covariates, higher CONUT remained independently associated with adverse outcome (OR 1.65, 95% CI 1.12–2.45 per 1-SD increase; *p* = 0.01). The association was similar in a sensitivity analysis excluding PaO_2_/FiO_2_ (OR 1.79, 95% CI 1.28–2.49; *p* < 0.001). **Conclusions**: A higher CONUT score at admission was independently associated with an increased risk of in-hospital death or ICU transfer, supporting its prognostic relevance as an immuno-nutritional index in hospitalized patients with COVID-19.

## 1. Introduction

Malnutrition is common among hospitalized patients and represents an important determinant of vulnerability to acute illness [1]. Poor nutritional status may impair immune competence and reduce metabolic and functional reserves, while acute illness and inflammation further promote anorexia, metabolic alterations, and muscle catabolism [2,3]. Accordingly, nutritional risk has been associated with longer hospital stay, complications, and mortality across different clinical settings. During the COVID-19 pandemic, these issues became particularly relevant, as severe SARS-CoV-2 infection frequently occurred in older individuals and in patients with multiple comorbidities and varying degrees of functional impairment [4,5]. The relationship between nutritional status and COVID-19 outcomes is, however, complex. Obesity and excess adiposity were identified early in the pandemic as risk factors for severe COVID-19, hospitalization, and mortality [6,7,8]. At the same time, patients admitted to hospital with severe disease may be particularly vulnerable to the consequences of inadequate nutritional reserves, frailty, and disease-related catabolism. Thus, anthropometric characteristics and nutritional status may provide complementary information when assessing prognosis in patients who have already developed severe disease. Several approaches have been proposed to assess nutritional risk in hospitalized patients. In addition to anthropometric measures, laboratory-based indices may be particularly useful when conventional nutritional assessment is difficult to perform. This was especially relevant during the first phases of the COVID-19 pandemic, when isolation procedures, clinical workload, and the severity of patients’ conditions often limited the feasibility of comprehensive nutritional assessment. Among available indices, the Prognostic Nutritional Index (PNI), based on serum albumin and lymphocyte count [9], and the Controlling Nutritional Status (CONUT) score [10], which additionally incorporates total cholesterol, can be calculated from routinely collected laboratory data and have been investigated as markers of nutritional risk and prognostic indicators in hospitalized patients, including those with COVID-19.

The MODIeCOSe in Italia project collected clinical, anthropometric, and laboratory data from patients hospitalized with COVID-19 in several Italian centers during the first two pandemic waves [11]. The availability of different indicators related to nutritional status offered the opportunity to explore their relationship with subsequent clinical course within a large multicenter cohort. The present study therefore aimed to investigate the association between nutritional status at hospital admission, assessed by anthropometric measures and laboratory-derived nutritional indices, and clinical outcomes in patients hospitalized with COVID-19.

## 2. Methods

### 2.1. Study Design and Population

MODIeCOSe in Italia is a multicenter retrospective registry of adult patients hospitalized with confirmed SARS-CoV-2 infection in several Italian cities during the first two pandemic waves from 1 February 2020 to 31 January 2021. The study design and characteristics of the registry have been previously described [11]. The registry was coordinated by the Unit of Clinical Nutrition, Obesity and Metabolic Diseases, University Hospital Policlinico “P. Giaccone”, Palermo, Italy.

The original database included 1593 patients from 10 participating centers. One center (n = 397) was excluded from the present analysis because approximately 75% of patients had missing outcome and baseline data. Among the remaining 1196 patients, outcome data were unavailable for nine, resulting in a final study population of 1187 patients from nine centers.

The study was approved by the Ethics Committee “Palermo I” of the University Hospital Policlinico “P. Giaccone”, Palermo, Italy (ref. 11/2020, 18 December 2020). In the study documentation approved by the Ethics Committee, written informed consent was indicated as not applicable because of the observational and retrospective nature of the study. The study was registered in the ISRCTN registry (ISRCTN27707558).

### 2.2. Outcome Definition

The primary outcome was a composite of in-hospital death or transfer to an intensive care unit (ICU). Patients experiencing either event were classified as having an adverse outcome, whereas patients discharged alive without either event were classified as having a favorable outcome.

### 2.3. Data Quality Control

Before analysis, baseline clinical and laboratory variables underwent systematic range, plausibility, and unit-consistency checks. Laboratory measurements reported using different units across participating centers were harmonized. Values considered physiologically implausible or attributable to evident recording or decimal-point errors were either corrected when the intended value could be reliably identified or otherwise treated as missing. FiO_2_ values recorded as either fractions or percentages were standardized to a common format. A detailed variable-by-variable data-cleaning log was maintained throughout the analysis.

### 2.4. Nutritional Indices

PNI was calculated at hospital admission as 10 × serum albumin (g/dL) + 0.005 × absolute lymphocyte count (/mm^3^) [9]. CONUT score was calculated from serum albumin, total cholesterol, and absolute lymphocyte count according to the original scoring system [10], with the three components contributing 0–6, 0–3, and 0–3 points, respectively, for a total score ranging from 0 to 12. Higher CONUT scores indicate poorer nutritional status. Both indices were calculated only when all required components were available and passed the predefined plausibility checks; no imputation of missing components was performed.

### 2.5. Statistical Analysis

Continuous variables were compared between patients with adverse and favorable outcomes using the Mann–Whitney U test, whereas categorical variables were compared using the chi-square test. Variables associated with the outcome at *p* < 0.05 in univariate analyses were considered for multivariable modelling. To minimize collinearity, variables reflecting the same clinical domain or sharing components with the nutritional indices were not entered simultaneously in the same model.

A multivariable logistic regression model was constructed to evaluate the independent association between nutritional status and the primary outcome. The model included the CONUT score together with representative variables from clinically distinct domains that were associated with outcome in univariate analyses. Age and sex were included a priori irrespective of their univariate association with outcome. Nutritional indices not associated with the primary outcome in univariate analysis were not entered into multivariable models. Given the limited number of events (51 in the main model) in the complete-case dataset, additional covariates were selected among clinically relevant variables associated with the outcome in univariate analyses (*p* < 0.05), in order to maintain model parsimony and limit overfitting. This pragmatic approach resulted in the inclusion of functional autonomy, ischemic heart disease, and eGFR, representing distinct clinical domains of frailty, cardiovascular disease, and renal function, respectively. Given that respiratory impairment at admission represents an important marker of baseline disease severity and may influence both CONUT components and prognosis, PaO_2_/FiO_2_ was included in the main multivariable model. A sensitivity analysis excluding PaO_2_/FiO_2_ was also performed to assess the robustness of the association between CONUT and outcome to model specification. Total cholesterol is a component of the CONUT score and may be affected by lipid-lowering therapy, therefore, a further sensitivity analysis was performed by adding statin use to the core model. A further sensitivity analysis used cluster-robust standard errors grouped by center to account for potential within-center correlation. Continuous predictors were standardized and odds ratios (ORs) are therefore reported per 1-SD increase; binary predictors are reported as presence versus absence. Analyses were performed using complete cases for the variables included in each model. A two-sided *p* < 0.05 was considered statistically significant. Analyses were performed in Python version 3.10.12 using the statsmodels version 0.14.0, SciPy version 1.11.1, and pandas version 2.0.3 packages.

## 3. Results

The final study population included 1187 patients, of whom 275 (23.2%) experienced the composite adverse outcome of in-hospital death or transfer to the ICU, while 912 (76.8%) were discharged alive without either event.

Patients with an adverse outcome were older and more frequently showed impaired functional status and a greater burden of comorbidities than those with a favorable outcome. Renal function and respiratory status at admission were also poorer in patients who subsequently experienced an adverse outcome, as reflected by lower eGFR and PaO_2_/FiO_2_ values and a greater need for oxygen therapy (Table 1 and Supplementary Table S1).

Among the laboratory-derived nutritional indices, CONUT score was higher in patients with an adverse outcome than in those with a favorable outcome (4.4 ± 2.3 vs. 3.4 ± 1.8, *p* < 0.001), whereas PNI did not differ significantly between the two groups (46.2 ± 9.8 vs. 47.8 ± 8.8, *p* = 0.11).

Anthropometric measures also differed between outcome groups. Patients with an adverse outcome had lower body weight, BMI, waist circumference, and waist-to-height ratio than those with a favorable outcome. In particular, waist circumference was 91.7 vs. 98.6 cm (*p* = 0.002), and waist-to-height ratio was 0.5 vs. 0.6 (*p* = 0.001) in the adverse and favorable outcome groups, respectively (Supplementary Table S1).

### 3.1. Multivariable Analysis

**Figure 1:**
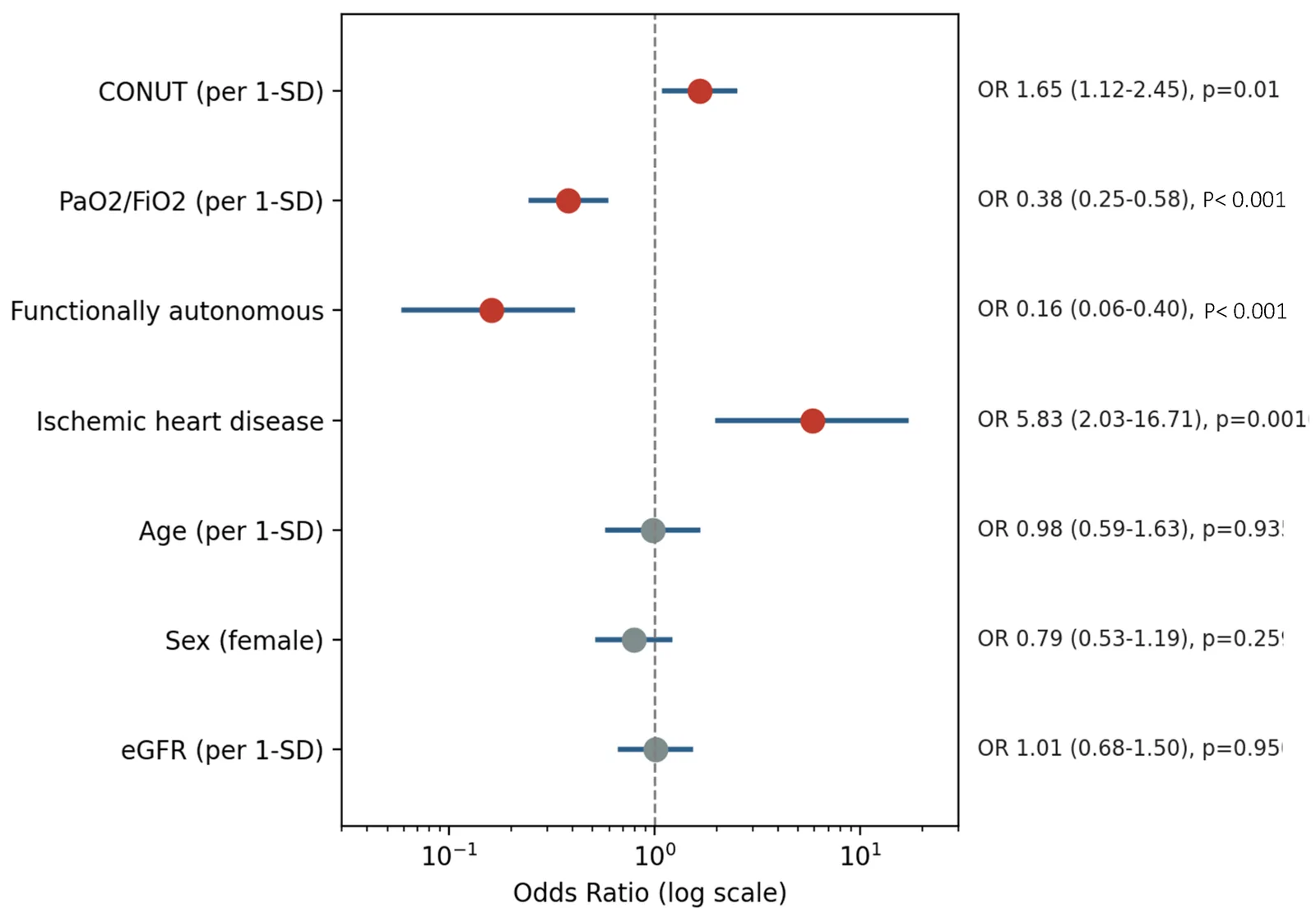
Main multivariable model, including admission PaO_2_/FiO_2_ (N = 230).

In the main multivariable model, additionally adjusted for respiratory impairment at admission, CONUT remained independently associated with adverse outcome (OR 1.65, 95% CI 1.12–2.45 per 1-SD increase; *p* = 0.01). PaO_2_/FiO_2_ was also independently associated with outcome (OR 0.38, 95% CI 0.25–0.58; *p* < 0.001), together with functional autonomy and ischemic heart disease. Age, sex and eGFR were not independently significant in this model (Figure 1; Table 1).

**Figure 2:**
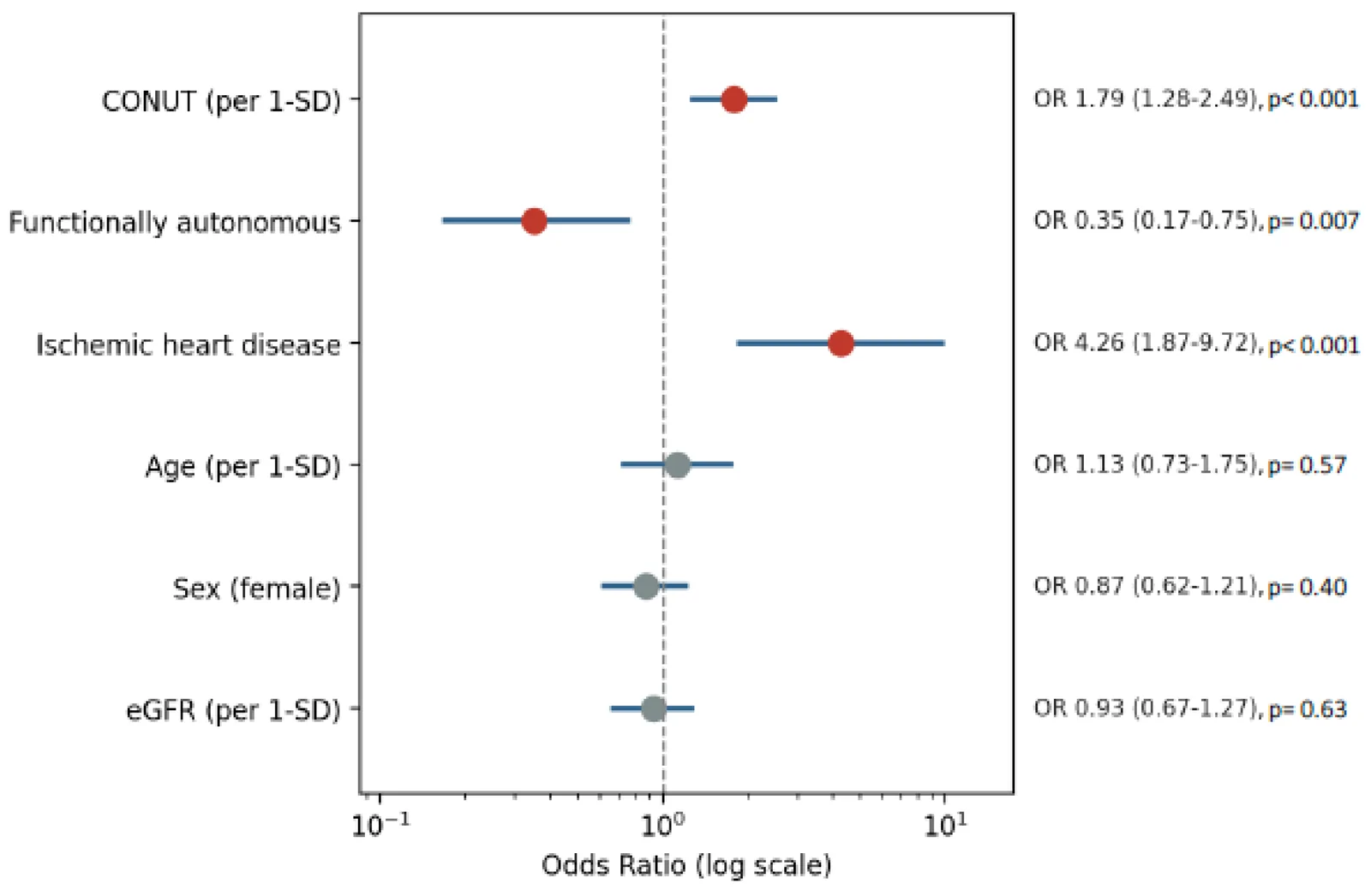
Sensitivity model excluding admission PaO_2_/FiO_2_ (N = 272).

In a sensitivity analysis excluding PaO_2_/FiO_2_, the association between CONUT and adverse outcome was similar (OR 1.79, 95% CI 1.28–2.49; *p* < 0.001) (Figure 2).

In a further sensitivity analysis including statin use, the association between CONUT and adverse outcome remained virtually unchanged (OR 1.84, 95% CI 1.30–2.60 per 1-SD increase, *p* < 0.001), whereas statin use was not independently associated with outcome (OR 1.50, 95% CI 0.70–3.22, *p* = 0.29). Functional autonomy (OR 0.35, 95% CI 0.16–0.76, *p* = 0.009) and ischemic heart disease (OR 4.02, 95% CI 1.66–9.73, *p* = 0.002) remained independently associated with outcome, whereas age, sex, and eGFR were not.

### 3.2. Sensitivity Analysis for Center Clustering

Complete cases for the sensitivity model excluding PaO_2_/FiO_2_ were concentrated in 5 of 9 centers (n = 111, 89, 54, 15 and 3 patients, respectively), reflecting heterogeneous completeness of nutritional and comorbidity data across the network; the three largest of these centers accounted for 254/272 patients (93%) of the sensitivity model sample. To account for potential within-center correlation, the sensitivity model excluding PaO_2_/FiO_2_ was refitted with cluster-robust standard errors grouped by center. CONUT remained statistically significant (OR unchanged at 1.79, cluster-robust *p* = 0.01, versus *p* < 0.001 with conventional standard errors) and ischemic heart disease remained highly significant (cluster-robust *p* < 0.001). Functional autonomy, in contrast, lost statistical significance under cluster-robust standard errors (*p* = 0.15, versus *p* = 0.007 with conventional standard errors), indicating that this specific association was more sensitive to the clustered, center-concentrated structure of the data than the CONUT association. Given the small number and marked imbalance of contributing centers, however, this cluster-robust analysis should be regarded as exploratory.

## 4. Discussion

In this multicenter cohort of patients hospitalized with COVID-19 during the first two pandemic waves in Italy, nutritional status at hospital admission was associated with subsequent clinical outcome. Three main findings emerged. First, a higher CONUT score was associated with an increased risk of the composite outcome of in-hospital death or ICU transfer after adjustment for relevant clinical variables, including the severity of respiratory impairment at admission; this association was consistent in the sensitivity analysis excluding PaO_2_/FiO_2_. Second, PNI was not significantly associated with outcome, suggesting that different laboratory-derived nutritional indices may not provide equivalent prognostic information in this clinical setting. Third, patients experiencing an adverse outcome had, on average, lower anthropometric measures of overall and abdominal adiposity, including body weight, BMI, waist circumference, and waist-to-height ratio. Although the latter findings should be interpreted cautiously because of the substantial amount of missing anthropometric data, taken together these observations suggest a complex relationship between nutritional status, body reserves, and prognosis during severe acute illness.

The association between higher CONUT score and adverse outcome is consistent with previous studies showing the prognostic relevance of impaired nutritional status in hospitalized patients with COVID-19 [4,5]. CONUT was originally developed and validated as a laboratory-based screening tool for hospital undernutrition and integrates serum albumin, total lymphocyte count, and total cholesterol [10]. In the setting of severe acute illness, however, CONUT should be interpreted as an immuno-nutritional rather than a purely nutritional index, since its components may be influenced by both nutritional status and the systemic inflammatory response. Notably, serum albumin, a negative acute-phase reactant, did not differ between outcome groups in our cohort, whereas lymphocyte count and total cholesterol were significantly lower in patients with adverse outcomes. Low cholesterol concentrations may accompany inadequate nutritional intake and undernutrition, although they may also be influenced by systemic inflammation and acute illness [12,13]. In our cohort, PNI, which includes albumin and lymphocyte count but not cholesterol, was not significantly associated with outcome, whereas CONUT was. This difference raises the possibility that the cholesterol component contributed additional information. However, this interpretation requires caution. Patients with an adverse outcome were more frequently treated with statins and had a higher prevalence of ischemic heart disease, which itself emerged as an independent predictor of adverse outcome. Thus, lower cholesterol concentrations in this group may partly reflect lipid-lowering treatment and cardiovascular comorbidity rather than nutritional status alone. However, in a sensitivity analysis including statin use, the association between CONUT and adverse outcome remained virtually unchanged, suggesting that it was not primarily explained by concomitant statin therapy. Given their different composition and weighting of individual components, CONUT and PNI may therefore capture partly different biological information in the setting of acute COVID-19; however, this interpretation remains hypothesis-generating and should not be considered evidence of superiority of CONUT over PNI.

The anthropometric findings provide an additional, although exploratory, perspective. Patients with an adverse outcome had lower body weight and BMI and, among those with available measurements, lower waist circumference and waist-to-height ratio and a lower prevalence of abdominal obesity. However, these comparisons were unadjusted and anthropometric data were available only in subsets of the cohort, with substantial and potentially non-random missingness. These findings should therefore be regarded as descriptive and hypothesis-generating and do not establish a protective effect of greater adiposity. Although this observation may appear to contrast with the well-established association between obesity and severe COVID-19, the relationship between adiposity and prognosis among patients already hospitalized with COVID-19 has been heterogeneous, particularly across different degrees of excess adiposity, whereas severe or extreme obesity appears more consistently associated with adverse outcomes [14,15]. Although COVID-19 no longer represents the epidemiological emergency existing at the time of this study, the present findings may have broader relevance to acute illness. Inflammation, reduced food intake, and increased catabolism are common features of severe acute diseases and may rapidly compromise nutritional reserves, particularly in older and vulnerable patients [2]. In this respect, the first two pandemic waves provided a unique clinical setting in which to investigate these relationships. The study population was entirely recruited before the introduction of COVID-19 vaccination, allowing the interaction between nutritional status, host response, and disease outcome to be examined without the potential modifying effect of vaccine-induced immunity. COVID-19 may therefore represent a useful clinical model for exploring the contribution of nutritional status and body reserves to the host response to severe acute illness.

This study has several limitations. Its retrospective multicenter design was associated with incomplete and heterogeneous availability of laboratory and, particularly, anthropometric data. Importantly, the multivariable analyses were restricted to complete cases and therefore included only a subset of the overall cohort (230 of 1187 patients in the main model and 272 in the sensitivity model excluding PaO_2_/FiO_2_), potentially introducing selection bias related to the non-random availability of clinical and laboratory data. The strong center-dependence of missing data may have further contributed to selection bias; moreover, the cluster-robust sensitivity analysis should be considered exploratory because of the small number and marked imbalance of contributing centers. Residual confounding cannot be excluded, particularly given the pragmatic covariate-selection strategy, the limited number of events available for multivariable modelling, and potential factors affecting individual CONUT components. Moreover, recent weight loss, dietary intake, body composition, and muscle mass or function were not available, limiting a more comprehensive characterization of nutritional status and preventing us from determining to what extent the lower body weight observed in patients with adverse outcomes reflected pre-existing or age-related malnutrition, the effects of acute infection, or their combination. Finally, ICU transfer may have been influenced by clinical and organizational factors during the exceptional circumstances of the first pandemic waves.

## 5. Conclusions

In this multicenter cohort of patients hospitalized with COVID-19, a higher CONUT score at admission was independently associated with an increased risk of in-hospital death or transfer to intensive care. Given that CONUT components may be influenced by both nutritional and inflammatory processes during acute illness, these findings support the prognostic relevance of CONUT as an immuno-nutritional index rather than demonstrating an independent effect of nutritional depletion itself. Exploratory anthropometric comparisons also showed lower measures of overall and abdominal adiposity among patients with adverse outcomes; however, given the substantial missingness and unadjusted nature of these analyses, these findings should be interpreted cautiously and considered hypothesis-generating. Prospective studies integrating immuno-nutritional assessment with comprehensive measures of body composition are warranted.

## Figures and Tables

**Table 1:** Multivariable logistic regression models for adverse outcome (death or transfer to intensive care).

Variable	Main Model OR (95%CI)	*p* (Main Model)	*p* (Cluster-Robust by Center)	Sensitivity OR (95%CI)	*p* (Sensitivity)
CONUT (per 1-SD)	1.65 (1.12–2.45)	0.01	0.01	1.79 (1.28–2.49)	<0.001
Functional autonomy	0.16 (0.06–0.40)	<0.001	0.15	0.35 (0.17–0.75)	0.007
Ischemic heart disease	5.83 (2.03–16.71)	0.001	<0.001	4.26 (1.87–9.72)	<0.001
Age (per 1-SD)	0.98 (0.59–1.63)	0.94	0.12	1.13 (0.73–1.75)	0.57
Sex (female)	0.79 (0.53–1.19)	0.26	0.64	0.87 (0.62–1.21)	0.40
eGFR (per 1-SD)	1.01 (0.68–1.50)	0.95	0.72	0.93 (0.67–1.27)	0.63
PaO2/FiO2 (per 1-SD)	0.38 (0.25–0.58)	<0.001	—		—

Main model n = 230 (51 events); sensitivity model n = 272 (60 events).

## Data Availability

The original contributions presented in this study are included in the article and its Supplementary Materials.

## Supplementary Materials

The following supporting information can be downloaded at https://www.mdpi.com/article/10.3390/nu18203378/s1, Table S1: Baseline characteristics by outcome group (adverse vs. favorable), MODIeCOSe in Italia registry.
