# Supplementary material for: Nutritional Status at Admission and Clinical Outcomes in Hospitalized Patients with COVID-19: Results from the MODIeCOSe in Italy Study
Source: Nutrients. 2026 Oct 15;18(20):3378. doi: 10.3390/nu18203378 (PMC13708032; doi:10.3390/nu18203378)
Supplement: Supplementary file 1 [file nutrients-18-03378-s001.zip › nutrients-4591048-supplementary.pdf]

Table S1. Baseline characteristics by outcome group (adverse vs. favorable), MODIeCOSe in Italia registry.

Variable	Adverse Outcome (n = 275)	Favorable Outcome (n = 912)	Available Cases (Adverse/Favorable)	*p*
Age (years)	74.8 ± 12.6	65.6 ± 14.5	275/911	<0.001
Male sex	70.5%	66.6%	275/912	0.24
Caucasian ethnicity	98.1%	94.7%	268/718	0.049
Currently employed	19.5%	45.5%	154/224	<0.001
Functionally autonomous	48.5%	79.1%	198/695	<0.001
Nursing home resident	26.3%	10.2%	198/694	<0.001
Has a caregiver	41.1%	18.2%	168/588	<0.001
Influenza vaccination	50.0%	38.3%	44/355	0.18
Pneumococcal vaccination	4.0%	8.2%	25/318	0.72
Height (m)	1.7 ± 0.1	1.7 ± 0.1	122/748	0.03
Weight (kg)	75.0 ± 17.6	78.6 ± 15.6	117/764	0.007
BMI (kg/m^2)	26.9 ± 6.4	27.5 ± 4.8	115/742	0.04
Waist circumference (cm)	91.7 ± 17.9	98.6 ± 13.9	42/342	0.002
Abdominal obesity	33.3%	52.0%	42/342	0.03
Waist-to-height ratio	0.5 ± 0.1	0.6 ± 0.1	42/342	0.001
GLP-1 agonists	1.3%	0.6%	230/702	0.50
DPP-4 inhibitors	0.4%	1.0%	230/702	0.69
SGLT-2 inhibitors	0.0%	1.1%	230/700	0.22
Insulin	10.4%	6.9%	231/699	0.11
Metformin	14.3%	12.4%	230/700	0.52
ACEI/ARBs	44.1%	37.4%	229/701	0.08
Diuretics	38.4%	21.7%	229/701	<0.001
Beta-blockers	35.7%	24.4%	230/701	0.001
Statins	31.1%	21.3%	228/701	0.003
White blood cells (10^9/L)	9.6 ± 5.3	8.1 ± 6.2	252/898	<0.001
Red blood cells (10^{12}/L)	4.4 ± 0.8	4.5 ± 0.7	248/707	0.87
Hemoglobin (g/dL)	12.9 ± 2.1	13.0 ± 1.8	255/897	0.30
Hematocrit (%)	38.8 ± 6.2	38.4 ± 5.1	244/704	0.30
MCV (fl)	87.6 ± 7.8	85.8 ± 8.4	241/703	<0.001
Platelets, (10^9/L)	221.8 ± 102.1	255.1 ± 106.0	253/896	<0.001
Lymphocytes, (10^9/L)	1.0 ± 1.0	1.1 ± 0.7	237/690	<0.001
Neutrophils, (10^9/L)	8.1 ± 4.9	6.5 ± 4.8	238/697	<0.001
Glucose, (mg/dL)	147.3 ± 82.5	122.8 ± 63.0	241/883	<0.001
HbA1c, (%)	10.3 ± 11.2	6.5 ± 1.5	37/219	0.93
Creatinine, (mg/dL)	1.4 ± 1.1	1.0 ± 0.7	252/887	<0.001
Total cholesterol, (mg/dL)	145.6 ± 43.9	157.9 ± 39.6	133/451	<0.001
HDL cholesterol, (mg/dL)	32.6 ± 13.4	33.9 ± 11.5	125/447	0.26
Triglycerides, (mg/dL)	159.8 ± 68.3	137.8 ± 66.2	142/476	<0.001
ALT, (U/L)	35.6 ± 34.2	43.4 ± 68.9	244/837	0.04
AST, (U/L)	61.2 ± 160.9	39.2 ± 51.2	245/827	0.001
GGT, (U/L)	91.7 ± 338.9	52.7 ± 59.6	220/774	0.75
Albumin, (g/dL)	4.1 ± 0.9	4.2 ± 0.8	192/422	0.60
Total protein, (g/dL)	6.3 ± 0.8	6.7 ± 0.7	190/575	<0.001
eGFR, admission	63.1 ± 33.0	81.0 ± 27.0	229/634	<0.001
IL-6, admission (pg/mL)	547.7 ± 4597.8	343.4 ± 586.6	118/452	0.02
CRP, admission (mg/dL)	34.5 ± 54.9	28.6 ± 41.1	245/836	0.01
CK, admission (mg/dL)	296.6 ± 452.9	141.8 ± 214.0	183/598	<0.001

LDH, admission (U/L)	510.4 ± 296.0	435.4 ± 214.0	212/636	0.001
Ferritin, (ng/mL)	1147.9 ± 1301.3	827.0 ± 918.3	151/734	<0.001
PaO_2	69.2 ± 24.2	75.9 ± 25.6	193/513	<0.001
$PaCO_2$	34.6 ± 5.9	34.5 ± 5.6	194/512	0.85
FiO_2	0.4 ± 0.2	0.3 ± 0.1	194/501	<0.001
PaO_2/FiO_2	205.1 ± 127.2	259.3 ± 135.6	183/651	<0.001
pH	7.4 ± 0.1	7.4 ± 0.2	194/512	<0.001
Lactate,	1.9 ± 1.5	1.4 ± 1.3	156/401	<0.001
$HCO3^-$,	23.4 ± 4.3	24.6 ± 4.9	184/482	0.002
SO_2, (%)	92.2 ± 8.0	94.9 ± 5.8	192/502	<0.001
Oxygen therapy	65.7%	41.9%	198/520	<0.001
PEEP	0.9 ± 2.9	0.1 ± 1.1	179/424	<0.001
Type 2 diabetes	27.7%	19.8%	242/910	0.01
Hypertension	67.2%	51.8%	241/910	<0.001
Ischemic heart disease	22.3%	8.4%	233/716	<0.001
Chronic heart failure	12.5%	7.1%	232/717	0.01
Smoking	11.2%	13.7%	206/685	0.43
Atrial fibrillation	14.1%	8.9%	234/710	0.03
Cancer	14.0%	9.8%	236/713	0.17
COPD	16.6%	8.8%	235/713	0.001
Asthma	3.8%	3.2%	234/711	0.81
Chronic kidney disease	21.6%	7.6%	236/714	<0.001
Stroke	9.4%	4.9%	234/713	0.02
Pulmonary embolism	3.4%	1.5%	234/714	0.13
Congenital heart disease	0.0%	0.7%	231/713	0.45
PNI	46.2 ± 9.8	47.8 ± 8.8	174/405	0.11
CONUT	4.4 ± 2.3	3.4 ± 1.8	97/243	0.006

Continuous 0. Mann–Whitney U test for continuous variables, chi-square test for categorical variables).
